# Effects of total sleep deprivation on divided attention performance

**DOI:** 10.1371/journal.pone.0187098

**Published:** 2017-11-22

**Authors:** Eric Chern-Pin Chua, Eric Fang, Joshua J. Gooley

**Affiliations:** 1 Center for Cognitive Neuroscience, Neuroscience and Behavioral Disorders Program, Duke-NUS Medical School, Singapore, Singapore; 2 Department of Diagnostic Radiology, Singapore General Hospital, Singapore, Singapore; National Taiwan University Hospital, TAIWAN

## Abstract

Dividing attention across two tasks performed simultaneously usually results in impaired performance on one or both tasks. Most studies have found no difference in the dual-task cost of dividing attention in rested and sleep-deprived states. We hypothesized that, for a divided attention task that is highly cognitively-demanding, performance would show greater impairment during exposure to sleep deprivation. A group of 30 healthy males aged 21–30 years was exposed to 40 h of continuous wakefulness in a laboratory setting. Every 2 h, subjects completed a divided attention task comprising 3 blocks in which an auditory Go/No-Go task was 1) performed alone (single task); 2) performed simultaneously with a visual Go/No-Go task (dual task); and 3) performed simultaneously with both a visual Go/No-Go task and a visually-guided motor tracking task (triple task). Performance on all tasks showed substantial deterioration during exposure to sleep deprivation. A significant interaction was observed between task load and time since wake on auditory Go/No-Go task performance, with greater impairment in response times and accuracy during extended wakefulness. Our results suggest that the ability to divide attention between multiple tasks is impaired during exposure to sleep deprivation. These findings have potential implications for occupations that require multi-tasking combined with long work hours and exposure to sleep loss.

## Introduction

Exposure to sleep deprivation impairs attention on simple tasks and increases distractibility [1–3]. However, it is often necessary to divide one’s attention between multiple activities, for example during driving or while multi-tasking at work. When more than one task is executed simultaneously, performance on one or both tasks is usually impaired [4]. This occurs because limited cognitive resources must be divided, resulting in mutual interference between tasks. The limits of attentional capacity are thought to vary with arousal level, with exposure to sleep deprivation resulting in greater difficulty in sustaining performance and effort [5]. Sleepy subjects can perform at normal levels when highly motivated, but this can give way to attentional lapses as homeostatic pressure to fall asleep competes with effort to remain awake [6].

In studies that have used a dual-task paradigm, it has been shown that performance on one or both tasks becomes worse during exposure to sleep deprivation [7–12]. To assess a person’s ability to divide attention, however, it is necessary to compare performance on a task performed alone versus in combination with at least one other task (i.e., between dual-task and single-task conditions). Most studies that have used this approach have found no difference in divided attention performance between rested and sleep-deprived states, including tasks that have combined visual search and mental arithmetic [13], verbal memory and mental arithmetic [14], visual and auditory target detection [15], simulated driving and mental arithmetic [16], and simulated driving and auditory reaction times [17].

Divided attention performance might be resistant to sleep deprivation as a consequence of greater cognitive engagement and reduced need for top-down cognitive control to sustain performance and motivation relative to simple attention tasks [2, 18]. Compensatory recruitment of attentional cognitive resources may also help to preserve divided attention performance during sleep deprivation, as suggested in some but not all brain imaging studies [14, 15]. For a divided attention task with high attentional demands, however, increased effort and recruitment of cognitive resources might be insufficient to compensate fully for the impairing effects of sleep deprivation on performance. To examine this possibility, divided attention was assessed using a complex task comprising single-task, dual-task, and triple-task conditions. We reasoned that it would be more challenging for subjects to divide their attention across three tasks compared with two tasks used in previous studies. Additionally, our divided attention task included visual and auditory tasks in which target stimuli were less frequent than non-targets, thus requiring sustained attention which is highly sensitive to sleep deprivation. We tested the hypothesis that effects of task load on response times and accuracy would be greater during exposure to total sleep deprivation compared with rested wakefulness, resulting in reduced ability to divide attention across multiple tasks in the sleep-deprived state.

## Materials and methods

### Subjects

Healthy males (n = 39) aged 21–30 years were enrolled in a 4-day study at the Chronobiology and Sleep Laboratory, Duke-NUS Medical School [19, 20]. Health was assessed by screening questionnaires and self-reported medical history. Subjects were ineligible if they had a history of shift work, or if they travelled across time zones within three weeks prior to the laboratory study. Subjects were also excluded if their chronotype score was <31 or >69 on the Horne-Östberg questionnaire [21], or if they experienced poor sleep quality in the month prior to the study (Pittsburgh Sleep Quality Index score >5) [22]. Participants were required to maintain a fixed sleep-wake schedule (8 hours sleep, 16 hours wake) of their choice for a week prior to the study, and this was verified by actigraphy monitoring (Actiwatch-L, MiniMitter, Inc., Bend, OR). During pre-study screening, subjects were asked to avoid caffeine, nicotine, alcohol, and over-the-counter medications. Informed written consent was obtained from all participants, and research procedures were approved by the SingHealth Centralized Institutional Review Board. The study complied with ethical principles described in the Declaration of Helsinki.

Of the 39 subjects who were enrolled in the laboratory study, 1 subject withdrew due to difficulty tolerating the sleep deprivation procedures. An additional 8 subjects were excluded post hoc, including 7 subjects in whom technical problems resulted in missing data, and 1 subject who did not comply with instructions for completing the divided attention task. Hence, 30 subjects (mean age ± SD = 25.8 ± 2.7 years) were included in the present analysis (S1 Dataset).

### Protocol

Subjects stayed individually for four days in a chronobiology research facility without access to natural light or other time cues. Participants arrived at the laboratory in the evening and were oriented to study procedures. The orientation included 2 practice sessions on the divided attention task (see below) to familiarize subjects with the task and minimize effects of learning on performance. Subjects went to bed at their regular pre-study sleep time. After 8 h of time in bed for sleep, subjects underwent a 40-h constant routine procedure consisting of wakefulness enforced by research staff, constant semi-recumbent position in bed, exposure to continuous dim light (approximately 0.6 lux at eye-level), and consumption of hourly equicaloric snacks. These procedures were implemented as part of a pair of studies that were designed to examine physiologic correlates of alertness and circadian regulation of plasma lipids. Results for self-rated sleepiness, sustained visual attention, circadian phase, the waking electroencephalogram (EEG), eye closures, heart rate variability, and plasma lipids are published elsewhere [19, 23–25]. Here, we report results only for the divided attention task, which was administered as a stand-alone task that alternated hourly with another test battery. After the constant routine procedure, participants were given an opportunity for recovery sleep (12 h in bed) before being discharged from the study. Research personnel were present throughout the study to carry out research procedures and to ensure protocol compliance.

### Divided attention task

Subjects completed a 15-min divided attention task every 2 h during the constant routine procedure, starting 3 h after their wake-up time. The task consisted of three 5-min blocks with increasing task load (Table 1). During the first block (single task), participants completed an auditory Go/No-Go (aGNG) task. During the second block (dual task), participants completed the same aGNG task while simultaneously performing a visual Go/No-Go (vGNG) task. In the third block (triple task), participants completed aGNG and vGNG tasks, while at the same time performing a motor tracking task. Each block was separated by a break that was self-paced but limited to 30 s. On the dual and triple tasks, participants were instructed to perform their best on all co-presented tasks, responding as quickly and accurately as possible for all target stimuli.

**Table 1:** Divided attention task with increasing task load across blocks.

Block	Duration	Auditory Go/No-Go	Visual Go/No-Go	Motor Tracking
**1. Single task**	5 min	Yes	No	No
**2. Dual task**	5 min	Yes	Yes	No
**3. Triple task**	5 min	Yes	Yes	Yes

The rationale for using Go/No-Go and motor tracking tasks is that cognitive processes required for these tasks (motor function, sustained attention, response inhibition, and working memory) are sensitive to the effects of total sleep deprivation and circadian phase [26–29]. In addition, pilot experiments established that these tasks were amenable to being performed simultaneously, with stable performance (>85% accuracy) on single, dual, and triple tasks and no evidence of learning during repeated daytime testing (every 2 h). The divided attention task was administered using E-prime 2.0 Professional software (Psychology Software Tools, Inc., Sharpsburg, PA) on a liquid crystal display (LCD) monitor with screen resolution of 1920 pixels by 1080 pixels. Details are provided below for aGNG, vGNG, and motor tracking tasks.

#### Auditory Go/No-Go task

A series of recorded numbers (1 to 6) were read aloud from desktop speakers, with a fixed inter-stimulus interval of 3 s. Subjects were instructed to press the middle button of a response box with their left middle finger as quickly as possible whenever an even number (2, 4, or 6) was called, and to avoid responding whenever an odd number (1, 3, or 5) was called. To ensure that participants could not predict reliably when the next auditory stimulus would occur, a subset of trials consisted of silence in which no number was called. Each 5-min aGNG task consisted of 30 even numbers (Go stimuli), 30 odd numbers (No-Go stimuli), and 40 silent trials, with the order of presentation randomized (Table 2). The duration of each auditory stimulus (or period of silence) was 0.5 s. The proportion of Go and No-Go stimuli on the aGNG task were the same for single, dual, and triple tasks. While performing the aGNG task in the single-task condition, participants were instructed to fixate on a small cross that was presented continuously in the center of the LCD screen.

**Table 2:** Frequency of stimuli in auditory and visual Go/No-Go tasks.

Stimulus	Auditory Go/No-Go		Visual Go/No-Go	
	Frequency	Sound	Frequency	Tile color
**Go**	30%	2, 4, 6	25%	Orange
**No-Go**	30%	1, 3, 5	75%	Red, Blue, Green
**None**	40%	Silence

#### Visual Go/No-Go task

During the vGNG task, a square target was shown in the center of the screen. The target changed color every 3 s, while participants simultaneously completed the aGNG task. Subjects were instructed to press a button on a trackball using their right thumb as quickly as possible every time that the color of the target changed to orange, and to avoid pressing the button when the target changed to red, blue, or green. Each 5-min vGNG task consisted of 25 orange trials (Go stimuli) and 75 non-orange trials (No-Go stimuli), with the order of presentation randomized (Table 2). The proportion of Go and No-Go stimuli on the vGNG task were the same for dual and triple tasks. The onset of color changes on the vGNG task was timed to coincide with the onset of auditory stimuli (or silent trials) given during the aGNG task.

#### Motor tracking task

The square tile that served as the visual stimulus for the vGNG task became a moving target. Participants were instructed to track the moving square with a cursor controlled by a trackball with their right fingers (excluding the thumb), while at the same time performing aGNG and vGNG tasks. The target moved at a constant rate in a straight diagonal line in one of four possible directions (± 45° relative to vertical and horizontal planes). The motion path was randomized every 1 to 6 s, or when the target reached a boundary of the LCD screen.

### Data analysis and statistics

The time-course of performance (reaction time, response errors, and motor tracking) was examined for single (aGNG), dual (aGNG and vGNG), and triple tasks (aGNG, vGNG, and motor tracking task). In GNG tasks, reaction times were assessed only for correct responses to Go stimuli that occurred before the onset of the next trial (i.e., within 3 s of stimulus onset). Errors were determined as the sum of trials in which participants failed to respond to a Go stimulus (false negative), or incorrectly responded to a No-Go stimulus (false positive). In the motor tracking task, we determined the percentage of time that subjects successfully maintained the cursor on the moving target.

Repeated-measures ANOVA was used to test for interaction and main effects of task load (single, dual, & triple tasks) and time since wake (18 time points) on aGNG performance (reaction time and response errors). In separate analyses, ANOVA was used to examine effects of task load (dual & triple tasks) and time since wake on vGNG performance. Planned comparisons (*F*-tests) were performed, and Tukey's Honestly Significant Difference (HSD) test was used for multiple comparisons. For all tests, *P* < 0.05 was considered statistically significant. Statistical tests were performed using SigmaPlot 11.0 software (Systat Software Inc, San Jose, CA).

## Results

**Fig 1:**
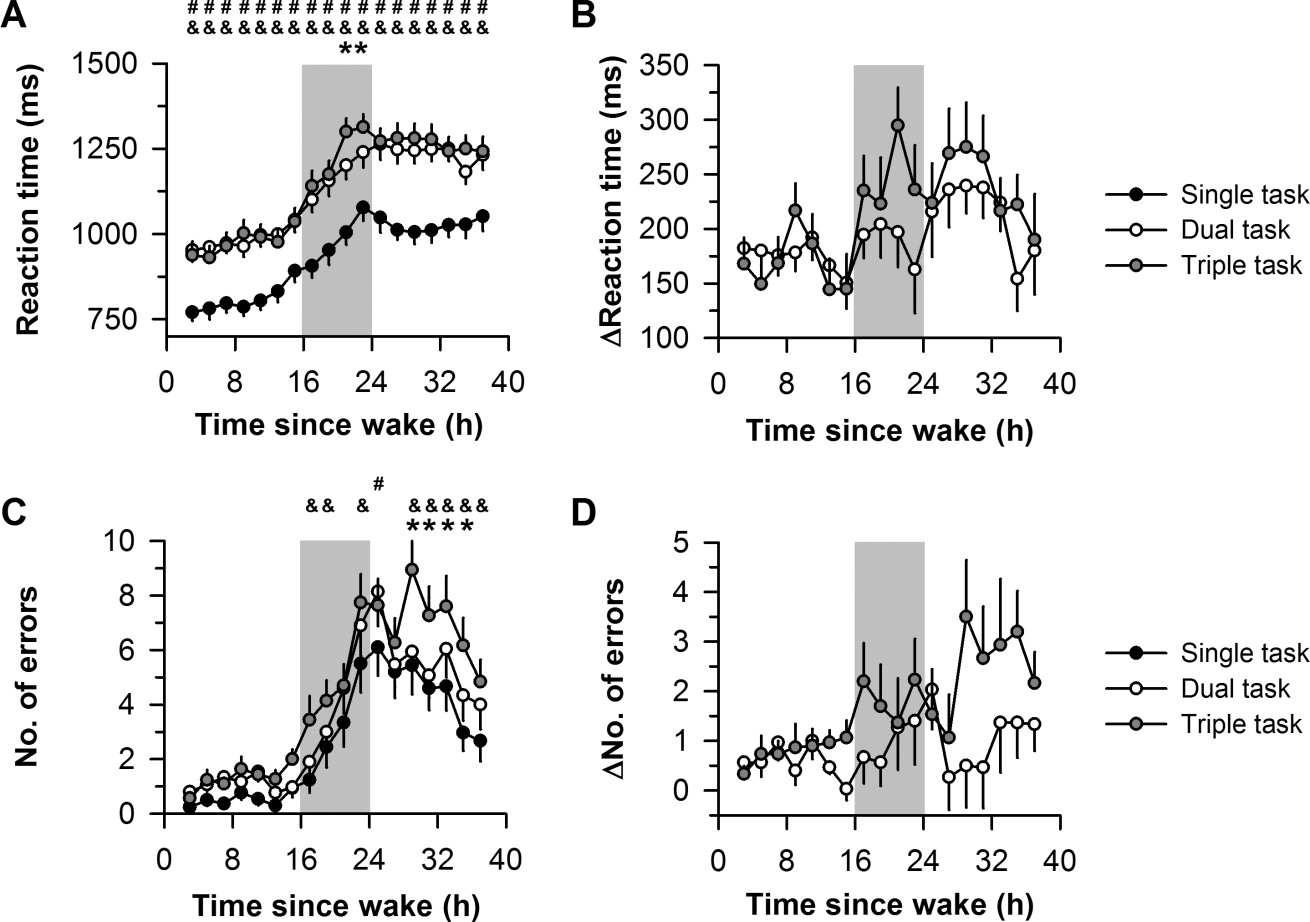
Effects of sleep deprivation on divided attention performance. Subjects completed (1) a single task consisting of an auditory Go/No-Go (aGNG) task, (2) a dual task in which the aGNG task was taken while simultaneously performing a visual Go/No-Go (vGNG) task, and (3) a triple task which included the aGNG and vGNG tasks, as well as a motor tracking task in which participants were required to maintain a cursor on a moving target. (**A**) The time course of reaction times on the aGNG task is shown during 40 h of sustained wakefulness for single (black circles), dual (white circles) and triple (gray circles) task conditions. (**B**) The increase in aGNG reaction times is shown during dual-task and triple-task conditions relative to the single-task condition. (**C**) The time course of errors on the aGNG task is shown during single, dual, and triple task conditions. (**D**) The increase in the number of errors is shown during dual-task and triple-task conditions relative to the single-task condition. The vertical gray box in each plot shows the usual hours of sleep. In panels **A** and **C**, hash marks (#) show significant differences in performance between single and dual tasks; ampersands (&) show significant differences between single and triple tasks; and asterisks (*) show significant differences between dual and triple tasks. In each plot, the mean ± SEM is shown.

A significant interaction was observed between task load and time since wake on aGNG response times (*F*_34,986_ = 2.00, *P* < 0.001), in which the impairing effects of increasing task load were greater during exposure to sleep deprivation (Fig 1A and 1B). Response times on the aGNG task were slower on dual and triple tasks compared with the single task irrespective of time since wake (Tukey’s HSD, *P* < 0.05 for all time points). By comparison, response times were slower on the triple task compared with the dual task only when subjects were sleep-deprived (Tukey’s HSD, *P* < 0.05 at 21 h and 23 h after wake-up time). Comparable results were obtained for response errors on the aGNG task (Fig 1C and 1D), in which increasing task load resulted in more errors only after subjects were kept awake beyond their usual bedtime (*F*_34,986_ = 1.76, *P* = 0.005 for the interaction; Tukey’s HSD, *P* < 0.05 at 17 h, 19 h, 23 h, 25 h, 29 h, 31 h, 33 h, 35 h, and 37 h after wake-up time). Secondary analyses demonstrated that errors on the aGNG task were predominantly false negatives (78.6% of all errors; failure to respond to a Go stimulus). A significant interaction was observed between task load and time since wake on false negative errors (*F*_34,986_ = 1.70, *P* = 0.008 for the interaction; Tukey’s HSD, *P* < 0.05 at 17 h, 23 h, 25 h, 29 h, 31 h, 33 h, 35 h, and 37 h after wake-up time), but not false positive errors (*F*_34,986_ = 1.01, *P* = 0.44 for the interaction).

**Fig 2:**
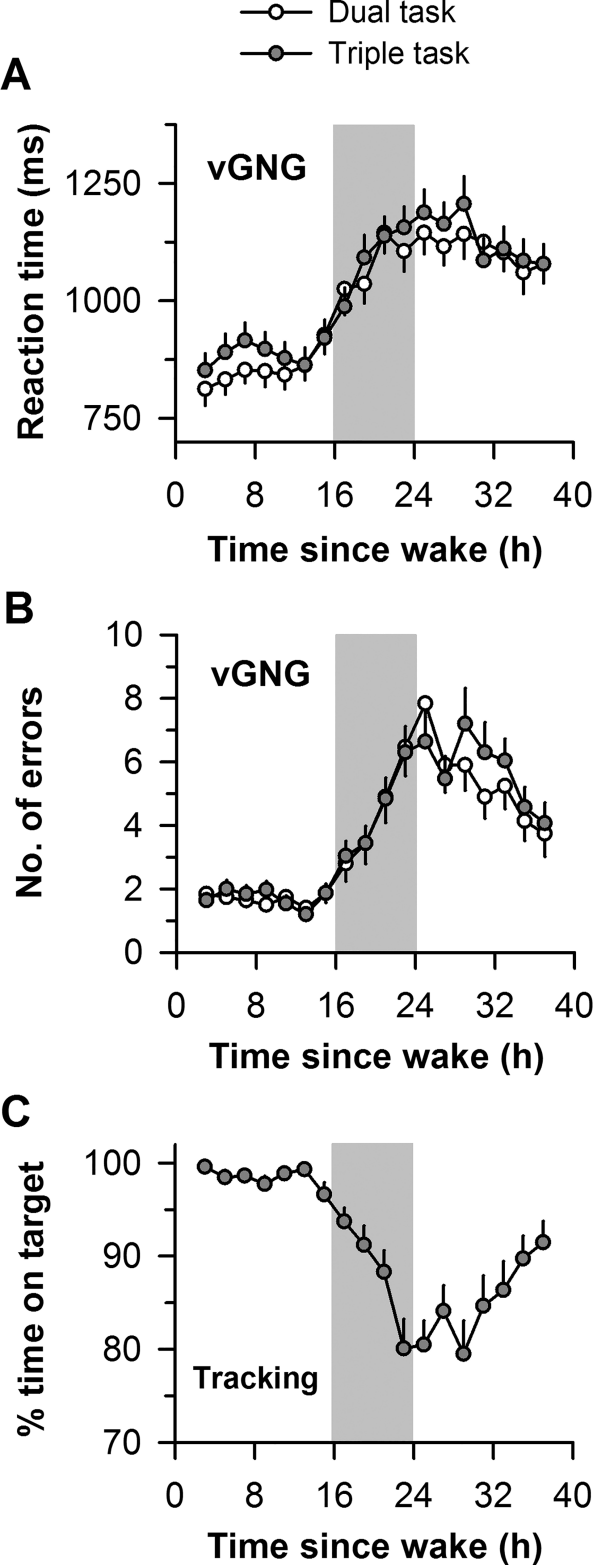
Effects of sleep deprivation on visual Go/No-Go and motor tracking performance. Subjects completed a dual task in which a visual Go/No-Go (vGNG) task was performed at the same time as an auditory Go/No-Go (aGNG) task, and a triple task that included aGNG and vGNG tasks, as well as a motor tracking task. The time course of (**A**) reaction times and (**B**) errors on the vGNG task is shown during 40 h of sustained wakefulness for dual (white circles) and triple (gray circles) task conditions. (**C**) The time course for tracking accuracy is shown for the motor tracking task in the triple-task condition. The vertical gray box in each plot shows the usual hours of sleep. In each plot, the mean ± SEM is shown.

Next, we determined whether performance on the vGNG task was modulated by task load and sleep deprivation by examining performance in dual-task and triple-task conditions (Fig 2A and 2B). There was no significant interaction between task load and time since wake on vGNG response times (*F*_17,493_ = 1.00, *P* = 0.45) or errors (*F*_17,493_ = 1.26, *P* = 0.22). There was no main effect of task load on response times (*F*_1,29_ = 1.45, *P* = 0.24) or errors (*F*_1,29_ = 0.66, *P* = 0.42), but there was a significant main effect of time since wake on both measures of vGNG task performance (Response times, *F*_17,493_ = 31.42, *P* < 0.001; Errors, *F*_17,493_ = 20.73, *P* < 0.001). Exposure to sleep deprivation also resulted in severe deficits in motor tracking performance, characterized by decreased tracking accuracy (Fig 2C).

## Discussion

We found that divided attention performance was impaired during exposure to total sleep deprivation, as demonstrated by a significant interaction between task load (single, dual, and triple tasks) and time since wake on aGNG response times and errors. As expected, performance on the aGNG task was poorer when subjects were required to divide their attention across additional tasks (i.e., vGNG and motor tracking tasks), and this effect was greater during exposure to sleep deprivation compared with rested wakefulness. Hence, effort and cognitive engagement were not enough to prevent the impairing effects of sleep deprivation. In addition, performance after usual bedtime deteriorated for all tasks that were co-presented (aGNG, vGNG, and motor tracking), indicating that multi-tasking performance was strongly impaired during exposure to sleep deprivation.

Most studies of divided attention have found that the dual-task cost (i.e., the difference in performance between dual-task and single-task conditions) was similar during exposure to sleep deprivation compared with rested wakefulness [13–17]. In those studies, it is possible that subjects had enough spare attentional capacity to maintain a stable dual-task cost during sleep loss. Similar to prior work, we found that the difference in aGNG performance between dual- and single-task conditions exhibited modest variation during prolonged wakefulness. In contrast, the triple-task condition helped to reveal the impairing effects of sleep loss on divided attention performance, perhaps as a consequence of greater mutual interference between tasks.

The primary outcome in our study was performance on the aGNG task, which was performed during each of the different task blocks (single, dual, and triple tasks). Go/No-Go tasks are commonly used to assess response inhibition, an important component of executive function [30]. Typically, Go events are presented at a higher frequency than No-Go events, hence creating a prepotent tendency to respond. In the present study, however, Go events were presented at a low frequency (30% for the aGNG task and 25% for the vGNG task). Hence, the wake-dependent decline in aGNG task performance that we observed may have been driven primarily by the effects of sleep deprivation on sustained attention [6, 31], rather than the effects of sleep loss on response inhibition and working memory [28, 31, 32]. Consistent with this interpretation, we observed that false negatives (failure to respond to Go stimuli) occurred nearly 4 times as often as false positives (responding to No-Go stimuli) on the aGNG task. Because our task assessed a limited number of cognitive processes, additional studies are needed to determine whether divided attention performance on other types of tasks and cognitive domains are similarly affected by sleep deprivation.

Recent studies suggest that divided attention performance may be circadian phase-dependent, with greater dual-task costs in the late subjective night [26, 33]. Because we observed decrements in performance throughout the period of extended wakefulness (i.e., from 16 h to 40 h after wake-up time), our results cannot be explained solely by circadian variation in divided attention performance. Nonetheless, performance on each of the tasks administered in our divided attention task appeared to be modulated by both circadian phase and homeostatic sleep pressure. Performance on each measure decreased during the usual hours of sleep, followed by partial improvement during the biological daytime. These results are similar to the time-course of drowsiness and simple attention performance reported previously using the same sleep deprivation procedures [24].

A limitation of our study is that we did not monitor sleep-wake state polysomnographically during the divided attention task. Therefore, we cannot distinguish between response errors caused by falling asleep versus those related to ‘zoning out’ or increased distractibility. Because task load was increased sequentially in our divided attention task, subjects were presented with different visual stimuli during single, dual, and triple tasks. An alternative approach would have been to present aGNG, vGNG, and motor tracking stimuli simultaneously in all blocks, and then instruct subjects to attend to only one, only two, or all three tasks. This would ensure that task parameters are identical across blocks [15], but also requires subjects to ignore irrelevant stimuli during single and dual tasks. The latter is potentially problematic for interpreting effects of cognitive load on divided attention because sleep deprivation impairs the ability to ignore task-irrelevant information [34]. In our study, the effects of sleep deprivation on distractibility were therefore minimized in the single task condition by presenting the aGNG task individually. This study design is similar to previous studies that have examined the effects of sleep deprivation on divided attention performance using single and dual task conditions [13, 16].

In the present study, subjects were instructed to perform their best on all co-presented tasks. In the triple task condition, however, the motor tracking task required subjects to follow the movements of the colored tile that also served as the visual stimulus in the vGNG task. This may have drawn subjects’ attention to the visual stimulus preferentially, hence explaining why there was no difference in performance on the vGNG task between dual and triple task conditions, whereas errors on the aGNG task increased during exposure to sleep deprivation. Because the order of task blocks was fixed with increasing cognitive load (single, dual, and triple task conditions), it is also possible that effects of order or time-on-task contributed to greater performance impairment on the aGNG task during the triple task.

Our finding that sleep deprivation impaired divided attention performance could have implications for safety-sensitive occupations that require shift work and long work hours. For example, air traffic controllers, military personnel, and health care workers must process multiple streams of information simultaneously while making moment-to-moment decisions affecting safety and health outcomes. However, the ability to sustain attention, ignore task-irrelevant information, and multi-task effectively is impaired during exposure to sleep deprivation [1, 34–36]. Fatigue and distraction, including use of handheld electronic devices, are also major contributors to driver crashes [37]. A multipronged approach may be necessary to reduce attentional failures associated with sleep loss and fatigue, including 1) interventions that aim to improve sleep behavior and work hours, 2) deployment of technologies that can be used to monitor and predict attentional failure, and 3) development of systems for automating tasks to reduce the need for multi-tasking. These approaches are important because our results suggest that effort and cognitive engagement are insufficient to prevent the impairing effects of sleep deprivation on multi-tasking performance.

## Supporting information

S1 Dataset Subject data for divided attention task. (XLSX)
